# Molecular Factors Underlying the Deposition of Intramuscular Fat and Collagen in Skeletal Muscle of Nellore and Angus Cattle

**DOI:** 10.1371/journal.pone.0139943

**Published:** 2015-10-05

**Authors:** Taiane S. Martins, Letícia M. P. Sanglard, Walmir Silva, Mário L. Chizzotti, Luciana N. Rennó, Nick V. L. Serão, Fabyano F. Silva, Simone E. F. Guimarães, Márcio M. Ladeira, Michael V. Dodson, Min Du, Marcio S. Duarte

**Affiliations:** 1 Department of Animal Science, Universidade Federal de Viçosa, Viçosa, Brazil; 2 Animal Biotechnology Laboratory—LABTEC, Universidade Federal de Viçosa, Viçosa, Minas Gerais, Brazil; 3 Department of Animal Science, North Carolina State University, Raleigh, North Carolina, United States of America; 4 Department of Animal Science, Universidade Federal de Lavras, Lavras, Brazil; 5 Department of Animal Science, Washington State University, Pullman, Washington, United States of America; Wageningen UR Livestock Research, NETHERLANDS

## Abstract

Studies have shown that intramuscular adipogenesis and fibrogenesis may concomitantly occur in skeletal muscle of beef cattle. Thus, we hypothesized that the discrepancy of intramuscular fat content in beef from Nellore and Angus was associated with differences in intramuscular adipogenesis and fibrogenesis during the finishing phase. To test our hypothesis, longissimus muscle samples of Nellore (*n* = 6; BW = 372.5 ± 37.3 kg) and Angus (*n* = 6; BW = 382.8 ± 23.9 kg) cattle were collected for analysis of gene and protein expression, and quantification of intramuscular fat and collagen. Least-squares means were estimated for the effect of Breed and differences were considered at *P* ≤ 0.05. A greater intramuscular fat content was observed in skeletal muscle of Angus compared to Nellore cattle (*P*≤0.05). No differences were observed for mRNA expression of lipogenic and lipolytic markers *ACC*, *FAS*, *FABP4*, *SERBP–1*, *CPT–2*, *LPL*, and *ACOX* (*P* > 0.05) in skeletal muscle of Nellore and Angus cattle. Similarly, no differences were observed in mRNA expression of adipogenic markers *Zfp423*, *PPARγ*, *and C/EBPα* (*P*>0.05) However, a greater PPARγ protein content was observed in skeletal muscle of Angus compared to Nellore cattle (*P*≤0.05). A greater abundance of adipo/fibrogenic cells, evaluated by the PDGFRα content, was observed in skeletal muscle of Angus than Nellore cattle (*P*≤0.05). No differences in fibrogenesis were observed in skeletal muscle of Angus and Nellore cattle, which is in accordance with the lack of differences in intramuscular collagen content in beef from both breeds (*P*>0.05). These findings demonstrate that difference in intramuscular fat content is associated with a slightly enhanced adipogenesis in skeletal muscle of Angus compared to Nellore cattle, while no difference in fibrogenesis.

## Introduction

Intramuscular fat (IMF) is one of the main factors that positively affect the perception of consumers of meat quality due to IMF influence on taste, juiciness, and tenderness [1]. However, enhancement of marbling without increasing the overall body fat accumulation is a challenge for the beef industry [2] and requires a deep investigation to understand the factors underlying IMF deposition.

In addition to IMF, intramuscular connective tissue is also a crucial meat quality trait known to negatively affect beef tenderness. Previous studies have shown that both adipocytes and fibroblasts share the immediate progenitor cells located in the evolving extracellular matrix of muscle fibers [3, 4]. Thus, improvement of marbling in beef may also lead to an enhancement of connective tissue deposition, which potentially compromises beef tenderness.

Fat deposition is a complex biological process under significant genetic control [5, 6], regulated by a large number of enzymes, hormones and metabolites regulating fat cell metabolism, deposition and differentiation. Consequently, the potential of IMF deposition varies among beef cattle breeds [1, 7, 8] and might be controlled by differences in intramuscular adipogenesis and fibrogenesis. Beef products derived from Nellore cattle are recognized by the international market as very lean meat due to the lack of intramuscular fat. Consequently, production strategies such as crossbreeding (*Bos indicus x Bos taurus*) have been adopted to improve quality of Nellore terminal-crossed beef [9]. However, no conclusive evidence is found regarding differences in intramuscular adipogenesis and fibrogenesis in Nellore skeletal muscle, compared to common *Bos taurus* breeds used in the beef industry. Therefore, we hypothesized that difference in intramuscular adipogenesis and fibrogenesis contributes to a discrepancy of IMF content in in skeletal muscle of Nellore and Angus cattle.

## Materials and Methods

### Ethical approval

All animal procedures were approved by the Animal Care and Use Committee of the Department of Animal Science from Universidade Federal de Viçosa, Brazil (19/2013-CEUAP).

### Animal handling, carcass data collection, and tissue sampling

A contemporary group of Nellore (*n* = 6; initial body weight = 372.5 ± 37.3 kg) and Angus (*n* = 6; initial body weight = 382.8 ± 23.9 kg) cattle with 20 months of age and raised under the same grazing conditions in a high quality *Brachiaria decumbens* pasture were confined in individual pens and fed *ad libitum* for a total of 84 days during the finishing phase. The feeding management used was chosen to be as representative as possible to the feeding conditions commonly observed in Brazilian beef systems. Chemical composition and ingredient proportion of the experimental diets are presented in Table 1.

**Table 1:** Ingredient proportion and chemical composition of experimental diet.

Item	
*Ingredient proportion*, *% of dry matter*	
Corn silage	30.0
Corn meal	58.0
Soybean meal	10.0
^1^Mineral mixture	2.0
*Chemical composition*, *%*	
Dry matter	72.0
Crude protein	12.4
Neutral detergent Fiber	26.2
Total digestible nutrients	78.1
Starch	49.7

^1^Mineral mixture: Ca = 45.0 g/kg; Mg = 7.5 g/kg; P = 11.0 g/kg; Cu = 104 mg/kg; Zn = 344 mg/kg; Se = 0.83 mg/kg; Virginiamycin = 140.0 mg/kg; Monensin = 120.0 mg/kg

To obtain the average daily gain data, cattle were weighed at the beginning and at the end of the experimental period and the body weight gain was divided by the days on feedlot. At the end of the finishing phase (84 days) all animals were harvested. Pre-harvest handling was in accordance with good animal welfare practices, and harvesting procedures followed the Brazilian Sanitary and Industrial Inspection Regulation for Animal Origin Products. After exsanguination, a sample of longissimus muscle (LM) was quickly collected from each animal and snap frozen in liquid nitrogen. Samples were then powdered in liquid nitrogen, placed in cryovials, and kept at -80°C until total RNA isolation and protein extraction. Another LM sample was collected after 24 h postmortem chill, freeze-dried, powdered, and kept at -20°C for further analysis of intramuscular collagen and fat.

After slaughter carcasses were chilled at 4°C for 24 h. At the end of the postmortem chill period, cold carcass weight (CCW), 12th rib fat thickness (RFT) and 12th rib longissimus muscle area (LMA) were measured on the left side of each carcass. Longissimus muscle areas were traced on transparencies and measured later with a planimeter and RFT measurements were taken ¾ the length ventrally over the longissimus muscle [10].

Carcasses were then weighed and separated into bone and soft tissue (lean and fat). Following carcass dissection, the soft tissues components (lean and fat) were ground using a bowl cutter. Carcass bones were first ground in an industrial grinder to reduce the bones into small particles, and then finally ground in a meat bowl cutter. Next, carcass tissues (lean, fat and bones) were homogenized in a meat bowl cutter and a composite sample was taken for each carcass and frozen (-80°C) for further chemical analyses.

### Total RNA isolation and quantitative real-time PCR (qPCR) analysis

Total RNA (1 μg) was extracted from 0.5 g of powdered tissue samples using Trizol® reagent (Invitrogen, Carlsbad, CA, USA). The RNA integrity (RIN) was evaluated through capillary electrophoresis using a RNA 6000 Nano kit and a 2100 Bioanalyzer System (Agilent Techonologies, CA, USA). Samples with RIN greater than 7.0 were treated with DNAse I, Amplification Grade (Invitrogen Carlsbad, CA, USA) and reverse transcribed into cDNA using the GoScript^TM^ Reverse Transcription System (Promega, Madison, WI, USA). The primer sets used are shown in Table 2. qPCR was performed on a 7300 Real-Times PCR System (Applied Biosystems) using GoTaq® kit from Promega and the following cycle parameters: 95°C for 3 min and 40 cycles at 95°C for 10 s and 60°C for 30 s. The amplification efficiency ranged from 0.90 to 0.99. After amplification, a melting curve (0.01 C/s) was used to confirm product purity. Gene expression values were calculated and expressed relative to GAPDH, as described by Livak and Schmittgen [11].

**Table 2:** List of primers.

Gene	Abbreviation	Forward sequence	Reverse sequence
Collagen type I, alpha 1	COL1A1	CCACCCCAGCCGCAAAGAGT	ACGCAGGTGACTGGTGGGATGTC
Collagen type III, alpha 1	COL3A1	GGCCCCCTGGAAAGGACGGA	CCCCGCCAGCACCACAACAT
Fibronectin 1	FN1	GCGTGTCACCTGGGCTCCAC	CGGTGCCGGGCAGGAGATTT
Transforming growth factor, beta 1	TGFβ-1	AGCCAGGGGGATGTGCCA	TAGCACGCGGGTGACCTCCT
Metalloproteinase–2	MMP2	CGTCGCCCATCATCAAA	CAGCCGTAGAAGGTGTTTAG
Tissue inhibitor of metalloproteinase–1	TIMP1	CCCAGCGCCCAGAGAGGCTA	TCTGTGGGTGGGGTGGGACG
Lysyl oxidase	LOX	AGCTCAGCATACGGGGAGA	CATCCATGCTGTGGTAATGC
CCAAT enhancer binding protein, alpha	C/EBPα	TGCGCAAGAGCCGGGACAAG	ACCAGGGAGCTCTCGGGCAG
Peroxissome proliferator activated-receptor gamma	PPARγ	TGGAGACCGCCCAGGTTTGC	AGCTGGGAGGACTCGGGGTG
Zinc finger protein 423	Zfp423	GGATTCCTCCGTGACAGCA	TCGTCCTCATTCCTCTCCTCT
Acetyl-CoA carbocylase	ACC	TGAAGAAGCAATGGATGAACC	TTCAGACACGGAGCCAATAA
Fatty acid synthase	FAS	ATCAACTCTGAGGGGCTGAA	CAACAAAACTGGTGCTCACG
Fatty acid binding protein 4	FABP4	GGATGATAAGATGGTGCTGGA	ATCCCTTGGCTTATGCTCTCT
Sterol regulatory element-binding protein 1	SERBP–1	GAGCCACCCTTCAACGAA	TGTCTTCTATGTCGGTCAGCA
Carnitine palmitoyltransferase 2	CPT–2	CATGACTGTCTCTGCCATCC	ATCACTTTTGGCAGGGTTCA
Lipoprotein lipase	LPL	CTCAGGACTCCCGAAGACAC	GTTTTGCTGCTGTGGTTGAA
Acyl-CoA oxidase	ACOX	GCTGTCCTAAGGCGTTTGTG	ATGATGCTCCCCTGAAGAAA
Glyceraldehyde 3-phosphate dehydrogenase	GAPDH	AGATAGCCGTAACTTCTGTGC	ACGATGTCCACTTTGCCAG

### Western blot analysis

Whole muscle protein was extracted in Lysis buffer (10mM Tris pH 7.2; 0.5% Triton X–100; 10% Glycerol; 0.5% Dithiothreitol-DTT; 0.5mM Phenylmethanesulfonyl fluoride-PMSF and 0.5mM Benzamidine). Protein content was measured through Bradford Protein Assay (Bio-Rad, Hercules, CA), and an equal amount of protein was separated through 10% SDS-PAGE. Proteins were transferred to nitrocellulose membranes and blocked with blocking solution (3% BSA w/v in TBSt) for 1 h with gentle agitation at room temperature. Membranes were then incubated with the following primary antibodies against to PPARγ (no. 2435S), PDGF Receptor α (no. 3174S), TGF-β (n. 3711S), β-tubulin (no. 2128) purchased from cell signaling (Danvers, MA, USA); ZFP–423 (no. SC48785) purchased from Santa Cruz Biotechnology (Dallas, TX, USA). Primary antibodies were incubated at 1:250 diluted in the blocking solution for 16 h at 4°C with gentle agitation. After incubation with primary antibodies, membranes were washed 3 times at room temperature with TBSt and then incubated with the appropriate horseradish peroxidase secondary antibody (goat anti-rabbit no. SC 2004 from Santa Cruz; goat anti-mouse no. A2554 from Sigma) at 1:5000 dilution, for 1 h at room temperature with gentle agitation. Then, membranes were washed 3 times (5 min each) with TBSt, developed with Clarity^TM^ ECL substrate (Bio-Rad, Hercules, CA), scanned with c-Digit® Blot scanner, and analyzed with Image Studio (Licor Biosciences). Band density of target proteins was normalized according to β-tubulin content.

### Collagen content analysis

Duplicate samples (3.0 g) from each powdered freeze-dried steak were hydrolyzed in 6N HCl at 105°C for 16h. Hydrolysates were filtered through #2 filter paper and diluted (1:500) in distilled water (dH_2_O). The pH of the filtered samples was adjusted to 6.0 with 2 N NaOH. Two ml of each of the neutralized filtrate was added to individual test tubes. To all tubes, 1 ml of an oxidant solution (1.41 g chloramine T dissolved in 100 ml of buffer solution, 30.0 g citric acid monohydrate, 15.0 g NaOH, 90 g sodium acetate trihydrate, dissolved in 500 ml dH_2_O then put in a 1 liter volumetric flask with 290 ml propanol) was added. After tubes had rested for 20 min at room temperature, 1 ml of color reagent (5g 4-dimethylbenzaldehyde, 20 ml propanol, 9 ml of 60% perchloric acid) was added to each tube. The samples were then vortexed, covered with aluminum foil and placed in a 60°C water bath for 15 min. Tubes were then removed, uncovered and allowed to cool to room temperature. Samples were then transferred to cuvettes and read in a spectrophotometer at 560 nm. Collagen content was calculated by multiplying hydroxyproline amount by 7.25 [12].

### Intramuscular fat content

Powdered freeze-dried muscle samples were analyzed for moisture by the Method 934.01 [13], and ether extract (EE) by the Method Am 5–04 [14], to determine the amount of intramuscular fat content.

### Carcass total fat content

All carcasses composite samples were frozen using liquid nitrogen and ground into powder using a blender. Carcass tissue samples were analyzed for moisture by the Method 934.01 [13], and ether extract (EE) by the Method Am 5–04 [14], to determine the amount of intramuscular fat content. Total fat mass was calculated by multiplying the percentage of EE and cold carcass weight.

### Statistical analysis

Data were analyzed using the fixed-effect model as follow:

$$Y_{ij}=\;\mu +B_{i}+IW_{\left(i\right)j}+e_{ij}$$

where *Y_ij_* is the phenotype of the individual; *μ* is the overall mean; *B*_*i*_ is the i^th^ level of the fixed effect of Breed; *IW*_*(i)j*_ is the fixed-effect of the covariate Initial Body Weight (iBW) within Breed; and *e*_*ij*_ is the random error associated with *Y*_*ij*_, distributed as $e_{i}{}_{j}\;∼N(0,I\sigma_{e}^{2})$.

Prior to the final analyses, the residuals from the analysis of each trait were assessed for normality using Shapiro-Wilk’s test. As expected, the gene expression data was not normal and it was transformed using *ln*(2^-ΔΔCt^ + 1) [15]. Once normality was met (*P* > 0.05), the effect of the covariate iBW within Breed was tested and removed from the final analyses when *P* > 0.10. Least-squares means were estimated for the effect of Breed. All analyses were performed using SAS 9.3 (Statistical Analysis System Institute, Inc., Cary, NC, USA).

## Results

None of the observations were considered outliers (*P* > 0.05), and thus, all data were used for the final statistical analysis. The effect of iBW within breed was not significant (*P* > 0.10) to all traits, and thus excluded from the final model.

### Average daily gain and carcass traits of Angus and Nellore cattle

Angus cattle had an average daily gain 37.3% greater (*P* < 0.01) than Nellore cattle. However, no differences were observed for cold carcass weight (*P* = 0.16) among breeds. Similarly, no differences were observed for longissimus muscle area (LMA; *P* = 0.39) neither for carcass rib fat thickness (RFT; *P* = 0.41) among Angus and Nellore cattle. Indeed, no differences were observed for carcass total fatness (CTF; *P* = 0.31) among breeds (Table 3). However, it should be noted that the number of observations does not allow definitive conclusions about animal performance and the lack of differences among breed may be due to the limited number of animals per treatment.

**Table 3:** Performance and carcass traits of Angus and Nellore cattle.

Item	Angus	Nellore	SEM	*P* -value
	*n = 6*	*n = 6*		
Average daily gain, kg/d	1.88	1.18	0.12	< 0.01
Cold carcass weight, kg	313.92	285.62	13.44	0.16
Longissimus muscle area, cm^2^	84.8	76.5	6.62	0.39
Rib fat thickness, mm	5.97	5.25	0.59	0.41
Carcass total fatness, kg	45.43	44.30	2.48	0.31

### mRNA expression of lipid metabolism markers in skeletal muscle of Angus and Nellore cattle

**Fig 1:**
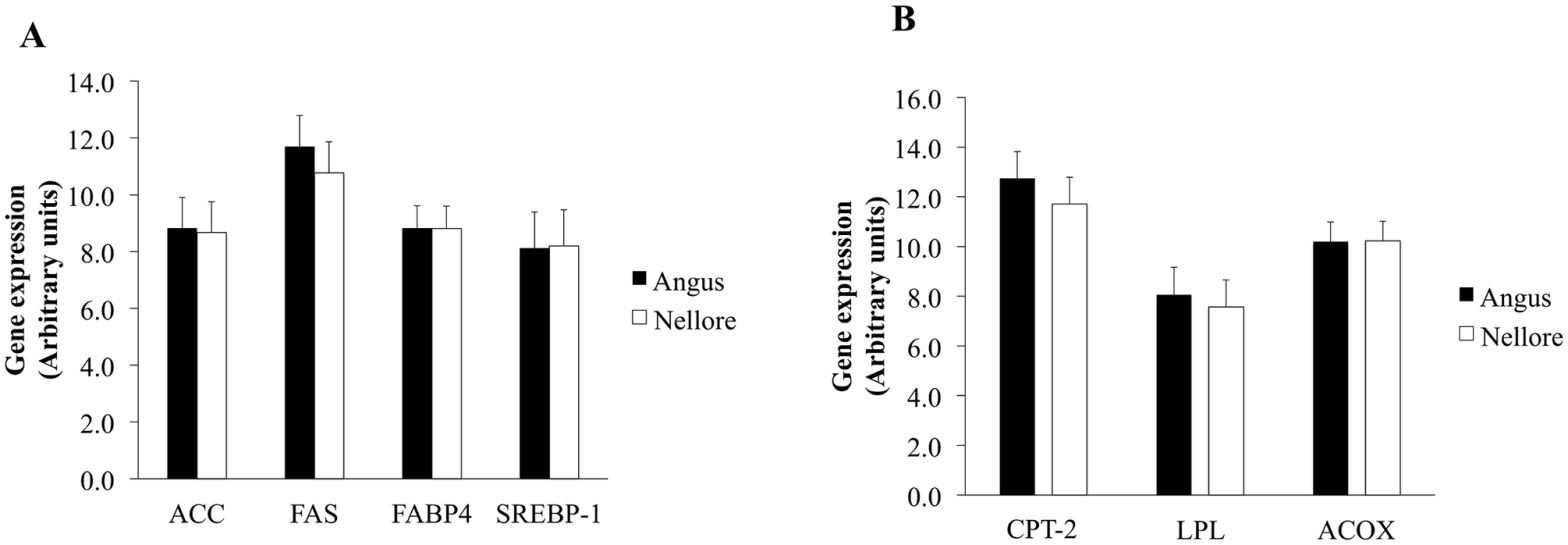
Expression of lipid metabolism markers in skeletal muscle of Angus and Nellore cattle. A) mRNA expression of *acetyl-CoA carboxilase* (*ACC*), *fatty acid synthase* (*FAS)*, *Fatty acid binding protein 4* (FABP4) *sterol regulatory element-binding protein 1* (*SERBP*-1); B) mRNA expression of *carnitine palmitoyltransferase 2* (*CPT–2*), *lipoprotein lipase* (*LPL*) *acyl-CoA oxidase* (*ACOX*). mRNA expression did not differ (*P* ≥ 0.05).

As adipose tissue deposition is caused by both adipogenesis and lipogenesis we investigated the mRNA expression of lipogenic markers. The abundance of mRNA of *acetyl-CoA carboxilase (ACC*; *P* = 0.82), *fatty acid synthase FAS* (*P* = 0.13), *fatty acid binding protein 4 (FABP4*; *P* = 0.99), and *sterol regulatory element-binding protein 1 (SERBP–1; P* = 0.91) did not differ among Angus and Nellore skeletal muscle (Fig 1A).

Similarly, no differences were observed for mRNA expression of lipid catabolism markers *carnitine palmitoyltransferase 2 (CPT–2*; *P* = 0.21), *lipoprotein lipase* (*P* = 0.50), *acyl-CoA oxidase* (*ACOX*; *P* = 0.98; Fig 1B).

### Intramuscular adipogenesis in skeletal muscle of Angus and Nellore cattle

**Fig 2:**
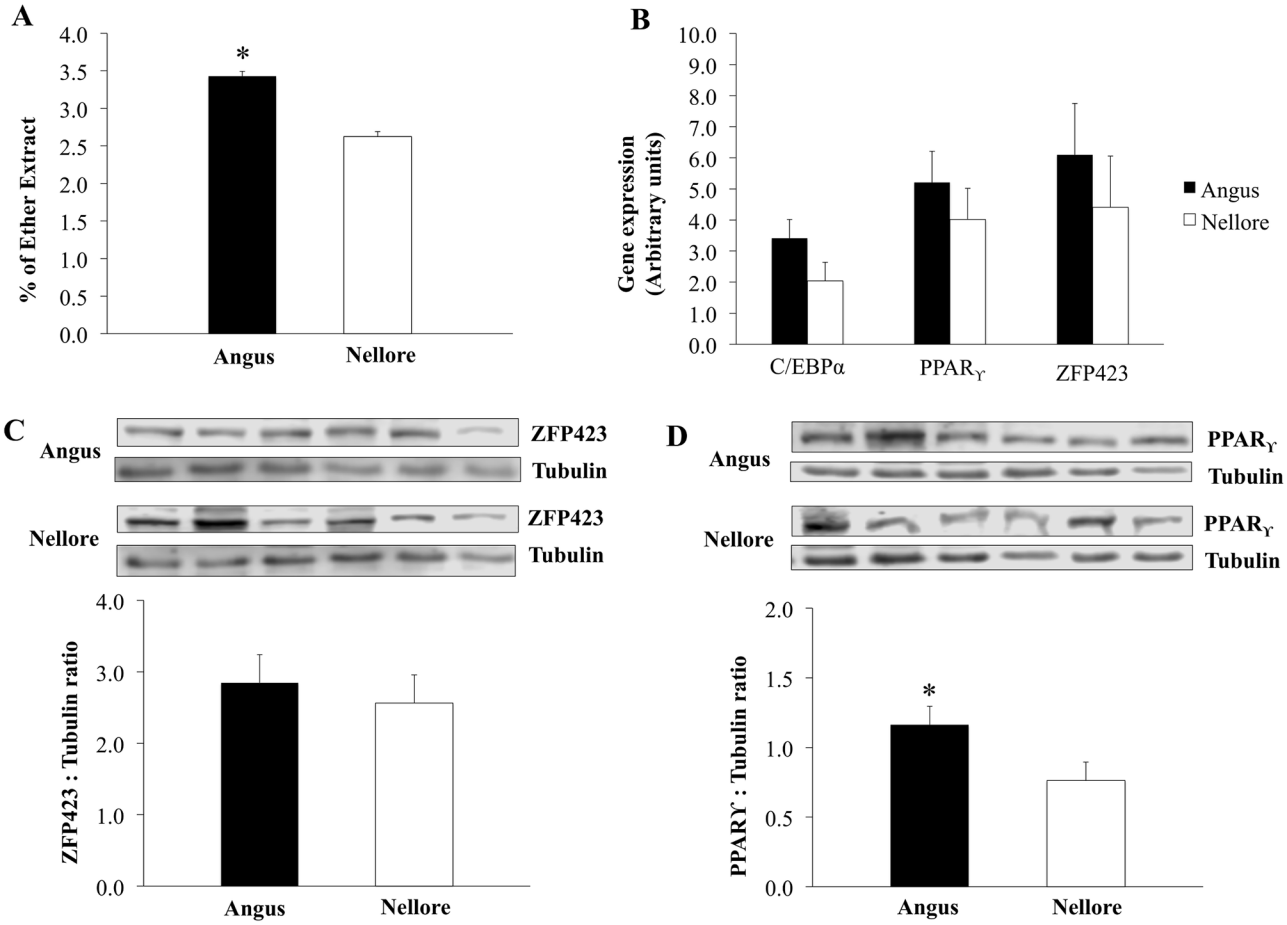
Intramuscular fat content and expression of adipogenic markers in skeletal muscle of Angus and Nellore cattle. Intramuscular fat measured by % of ether extract; B) mRNA expression of *CCAAT enhancer binding protein*, *alpha* (*C/EBPα*), *Peroxissome proliferator activated-receptor gamma* (*PPARγ*), and *Zinc finger protein 423* (*Zfp423*); C) Zfp423 abundance evaluated by western-blot; D) PPAR_ϒ_ abundance evaluated by western-blot; Tubulin was used as a loading control. mRNA expression and protein abundance did not differ (*P* ≥ 0.05).

A greater intramuscular fat content was observed in longissimus muscle of Angus compared to Nellore cattle (*P* < 0.01; Fig 2A). However, no differences were found for mRNA expression of late adipogenic markers *C/EBPα* (*P* = 0.14) and *PPARγ* (*P* = 0.42) in longissimus muscle of Angus and Nellore cattle (Fig 2B). Similarly, the mRNA expression of early adipogenic marker *Zfp423* in longissimus muscle did not differ (*P* = 0.62) among breeds (Fig 2B).

In agreement with mRNA observations, no differences were observed for protein levels of Zfp423 (*P* = 0.62; Fig 2C) while levels of PPARγ were greater (*P* = 0.05; Fig 2D) in longissimus muscle of Angus compared to Nellore cattle.

### Intramuscular fibrogenesis in skeletal muscle of Angus and Nellore cattle

**Fig 3:**
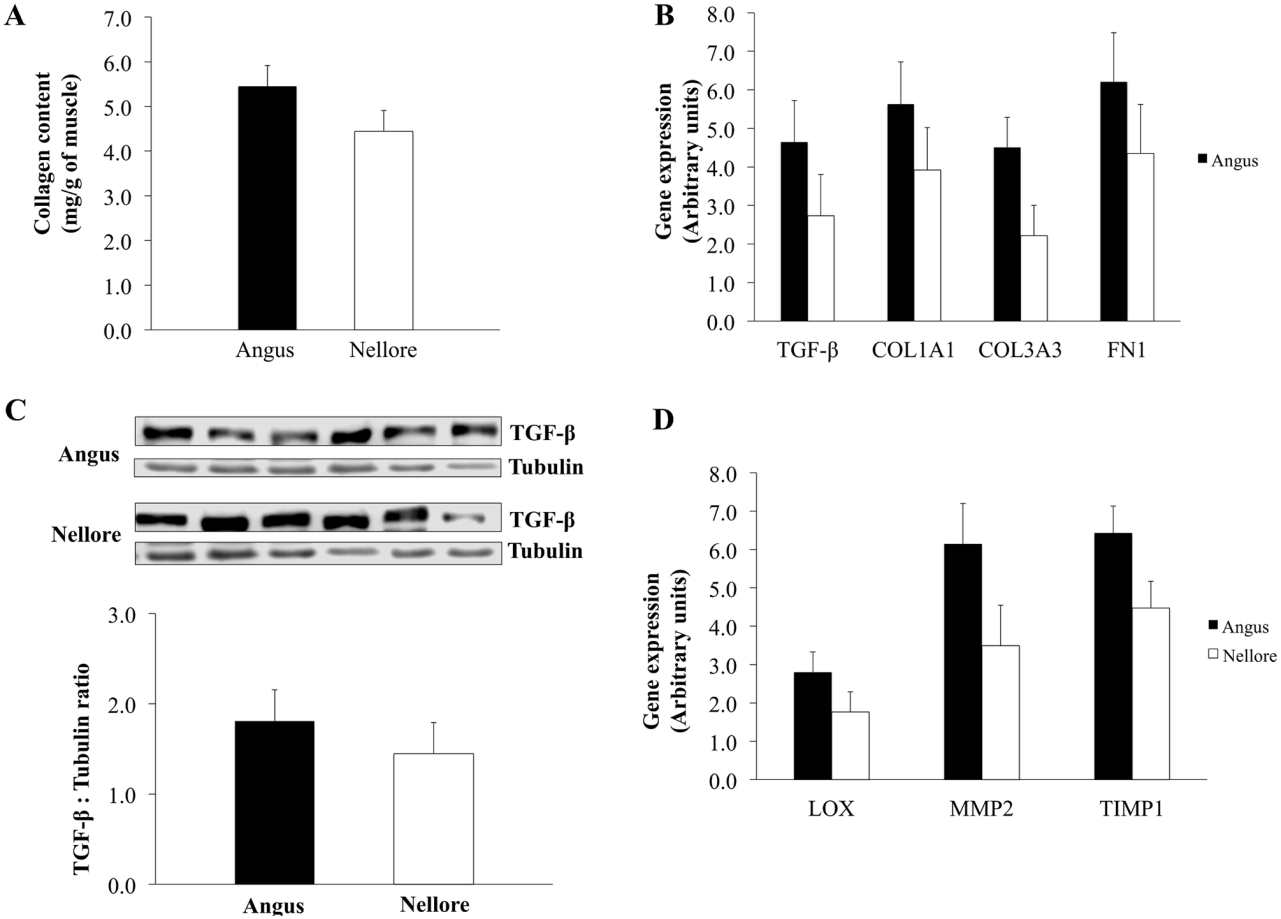
Collagen content, expression of fibrogenic and collagen remodeling markers in skeletal muscle of Angus and Nellore cattle. Intramuscular collagen content based on hydroxyproline concentration; B) mRNA expression of *transform growth factor beta* (*TGF-β*), *collagen I (COL1A1)*, *collagen III (COL3A3)*, and *fibronectin*; C) TGF-β abundance evaluated by western-blot; D) mRNA expression of *lysyl oxidase* (*LOX*), *metalloproteinase II* (*MMP2*) and *tissue inhibitor of metalloproteinase 1* (*TIMP1*); Tubulin was used as a loading control. Differences were considered at *P* ≤ 0.05 (*).

A similar content of total intramuscular collagen was observed in skeletal muscle of Angus and Nellore cattle (*P* = 0.16; Fig 3A). Indeed, no differences were observed for mRNA expression of fibrogenic markers *TGF-β* (*P* = 0.24), *collagen I* (*P* = 0.30), *fibronectin* (*P* = 0.32), and *collagen III* (*P* = 0.07) in skeletal muscle of Angus compared to Nellore cattle (Fig 3B).

Levels of TGF-β in skeletal muscle did not differ (*P* = 0.47; Fig 3C) between the two breeds. No differences were observed for mRNA expression of key enzymes of collagen remodeling *LOX* (*P* = 0.19), *MMP2* (*P* = 0.10), and *TIMP1* (*P* = 0.08) in skeletal muscle of Angus and Nellore cattle (Fig 3D).

### Identification of PDGFR_α_ abundance in skeletal muscle of Angus and Nellore cattle

**Fig 4:**
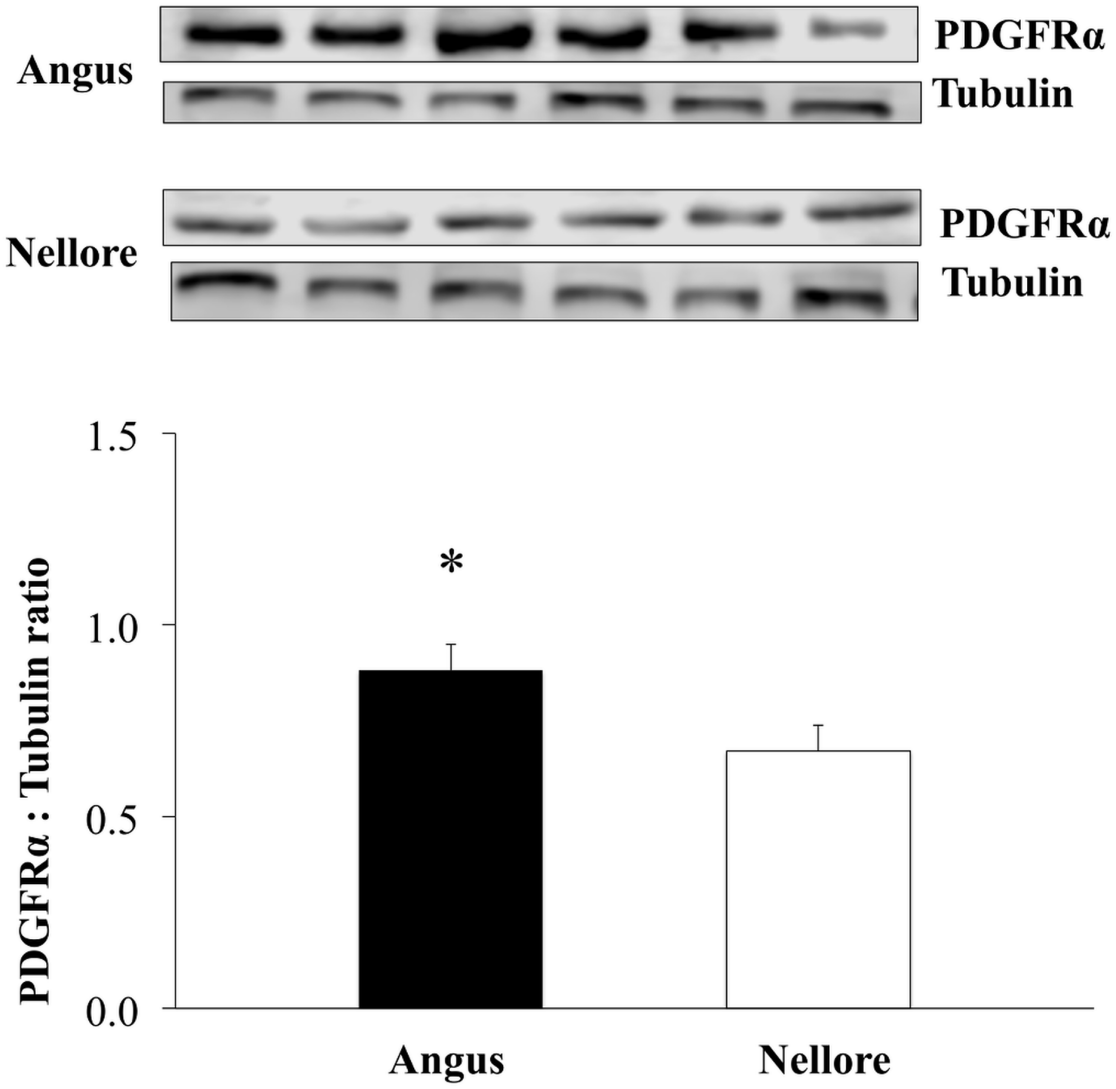
Abundance of PDGFR_*α*_ in skeletal muscle of Angus and Nellore cattle. Quantification of PDGFR_*α*_ by western-blot using tubulin as a loading control as an indicator of the abundance of mesenchymal progenitor cells. Differences were considered at *P* ≤ 0.05 (*).

To further understand the mechanisms underlying the collagen and fat deposition in skeletal muscle of meat animals we have evaluated the expression of PDGFR_α_, which is known as a marker for mesenchymal progenitor cells that can contribute to both fat and collagen deposition [3, 16]. The PDGFR_α_ content was greater in skeletal muscle of Angus compared to Nellore cattle (*P* = 0.05; Fig 4). Although the number of PDGFR_α_ positive cells was not evaluated in this study, the greater abundance of this protein may indicate a greater number of mesenchymal stem cell in skeletal muscle of Angus compared to Nellore cattle.

## Discussion

Differences in IMF content may vary as a consequence of intramuscular adipogenesis and/or lipogenesis. The former occurs as a consequence of adipocyte hyperplasia, which involves pre-adipocyte differentiation [17]. On the other hand, it can also be due to adipocyte hypertrophy without change in adipogenesis [1, 18, 19]. In this scenario changes in intramuscular lipogenesis may be mainly responsible for intramuscular fat deposition. In our previous study comparing IMF deposition in Wagyu and Angus skeletal muscle, we showed that enhancement of marbling in Wagyu was associated with elevated mRNA expression of both early (*Zfp423*) and late adipogenic markers (*PPAR*_*ϒ*_ and *C/EBP*_*α*_), indicating that enhanced adipogenesis was likely the driver of enhanced marbling in skeletal muscle of Wagyu compared to Angus cattle [8].

To evaluate potential differences in intramuscular lipogenesis we compared the mRNA expression of several genes encoding key proteins favoring lipid biosynthesis including *ACC*, *FAS*, *FABP4*, and *SERBP–1*. In the present study no differences in mRNA of any of theses markers were observed in skeletal muscle of Angus and Nellore cattle. Similarly, we have also evaluated the mRNA expression of several genes encoding key proteins favoring the lipid mobilization and fatty acid oxidation including *LPL*, *CPT–2*, and *ACOX*, which would also account for differences in the amount of intramuscular fat content among breeds. As a result, similar mRNA expression of lipolytic markers was observed in skeletal muscle of Angus and Nellore cattle. Although the activity of both lipogenic and lipolytic enzymes were not evaluated, these results suggest that intramuscular lipogenesis/lipolysis were not associated with differences in intramuscular fat content observed in the present study.

To further investigate the mechanisms underlying intramuscular fat deposition in beef cattle we hypothesized that differences in intramuscular adipogenesis may account for the discrepancy in marbling between Nellore (very lean-meat model) and Angus (moderate marbling model) cattle. Thus, we investigated if there were differences in early adipogenesis by comparing the mRNA and protein expression of *Zfp423* in skeletal muscle from both breeds as *Zfp423* is regarded as a key transcription factor responsible for early commitment of adipogenesis [5, 20, 21]. However, no differences in mRNA and protein expression of Zfp423 was observed in skeletal muscle of Angus and Nellore cattle, suggesting that differences in IMF content was not due to enhanced commitment of undifferentiated cells to the adipogenic lineage.

As differentiation of pre-adipocytes into adipocytes is mainly regulated by PPAR_ϒ_ and C/EBP_α_, we explored the expression of these late adipogenic markers. Despite the lack of differences in mRNA expression of *PPARγ* and *C/EBPα*, the PPAR_γ_ content was greater in skeletal muscle of Angus than Nellore cattle. PPAR_γ_ is a nuclear receptor that has been identified as the master regulator of adipogenesis [22] and its expression has been shown to be essential for the fat formation in vitro and in vivo [23]. Thus, several studies have evaluated the expression of PPAR_γ_ as marker for intramuscular fat content [1, 8]. Our results suggests an enhancement of adipogenic differentiation of progenitor cells of Angus muscle, which seems to a key factor that contributed to the greater IMF content in skeletal muscle of Angus compared to Nellore cattle.

Similar to IMF, intramuscular collagen content is also reported as an important factor of beef quality because it directly affects meat tenderness. Once intramuscular adipocytes and fibroblasts are developed from common progenitor cells [3, 4, 24], intramuscular adipogenesis and fibrogenesis may occur concomitantly leading to an enhancement of both intramuscular fat and collagen deposition in skeletal muscle [8]. Collagen synthesis is mainly mediated by TGF-β signaling pathway [25, 26], promoting fibrosis via activation of the SMAD signaling pathway [27, 28], which in turn induces the expression of fibrogenic genes, including fibronectin and type I collagen [29]. Therefore, the lack of differences for intramuscular collagen content between Angus and Nellore skeletal muscle was consistent with the similar mRNA expression of *TGF-β* and TGF-β contents in skeletal muscle of both breeds. Indeed, changes in mRNA expression of collagen remodeling markers, which would also account for differences in collagen content, were not observed in skeletal muscle of Angus and Nellore cattle. Together, our data shows no differences in fibrogenesis in skeletal muscle of Angus and Nellore cattle at the finishing phase.

Previous studies have shown the existence of mesenchymal progenitor cells in the skeletal muscle that presents the ability to commit to both adipogenic and fibrogenic lineages [3, 24]. Recently, these cells have been identified as residing in a muscle interstitium of mouse muscle by using a PDGFRα as a marker and showed that these cells directly contribute to intramuscular fat and connective tissue development [3, 4]. Additionally, PDGFRα was also shown to be an effective marker to identify mesenchymal progenitor cells in human skeletal muscle [16].

In the present study the expression of PDGFRα was evaluated by western blot analysis to test whether there is a difference in this protein abundance in skeletal muscle between the two breeds. Although the number of PDGFRα positive cells was not evaluated, protein abundance may indicate differences in adipo/fibrogenic cell abundance that would contribute to the formation of adipose and connective tissues in skeletal muscle of both breeds. We found a greater PDGFRα content in skeletal muscle of Angus compared to Nellore cattle, which might contribute to the slightly higher adipogenic differentiation detected in Angus muscle. The lack of difference in the expression of early adipogenic marker, Zfp423, and fibrogenic markers could be due to their lower expression in mature skeletal muscle. It will be very interesting to further examine the difference in adipogenesis and fibrogenesis of these two cattle breeds during early development, when adipogenesis and fibrogenesis are most active.

In conclusion, our findings demonstrate slightly enhanced adipogenesis in Angus compared to Nellore cattle muscle, while no difference in fibrogenesis. Although enzyme activity was not measured, our mRNA expression data of lipid metabolism markers suggests that intramuscular lipogenesis/lipolysis were not associated to differences in intramuscular fat content observed in the present study. We also found that the PDGFRα content was also higher in Angus, likely indicating enhanced progenitor density in Angus compared to Nellore cattle. Our data suggest that the difference in adipogenesis among breeds may have occurred due to a greater density of mesenchymal progenitor cells in Angus compared to Nellore skeletal muscle.

## Supporting Information

S1 Data Raw data according to experimental treatments. (XLSX)
